# Supplementary material for: Evaluation of the Drug-Induced Liver Injury Potential of Saxagliptin through Reactive Metabolite Identification in Rats
Source: Pharmaceutics. 2024 Jan 13;16(1):106. doi: 10.3390/pharmaceutics16010106 (PMC10819019; doi:10.3390/pharmaceutics16010106)
Supplement: Supplementary file 1 [file pharmaceutics-16-00106-s001.zip › pharmaceutics-2822130-supplementary.pdf]

Evaluation of the Drug-Induced Liver Injury Potential of Saxagliptin through Reactive Metabolite Identification in Rats

Ki-Young Kim [1,†], Yeo-Jin Jeong [1,†], So-Young Park [1,2], Eun-Ji Park [1], Ji-Hyeon Jeon [1], Im-Sook Song [1,*] and Kwang-Hyeon Liu [1,2,*]

[1] BK21 FOUR KNU Community-Based Intelligent Novel Drug Discovery Education Unit, Research Institute of Pharmaceutical Sciences, College of Pharmacy, Kyungpook National University, Daegu 41566, Republic of Korea; neanic12@naver.com (K.-Y.K.); duwls9902@gmail.com (Y.-J.J.); soyoung561@hanmail.net (S.-Y.P.); roseej98@naver.com (E.-J.P.); kei7016@naver.com (J.-H.J.)

[2] Mass Spectrometry Based Convergence Research Institute, Kyungpook National University, Daegu 41566, Republic of Korea

* Correspondence: isssong@knu.ac.kr (I.-S.S.); dstlkh@knu.ac.kr (K.-H.L.); Tel.: +82-53-950-8569 (I.-S.S.); +82-53-980-8567 (K.-H.L.); Fax: +82-53-950-8557 (I.-S.S. & K.-H.L.)

† These authors contributed equally to this work.

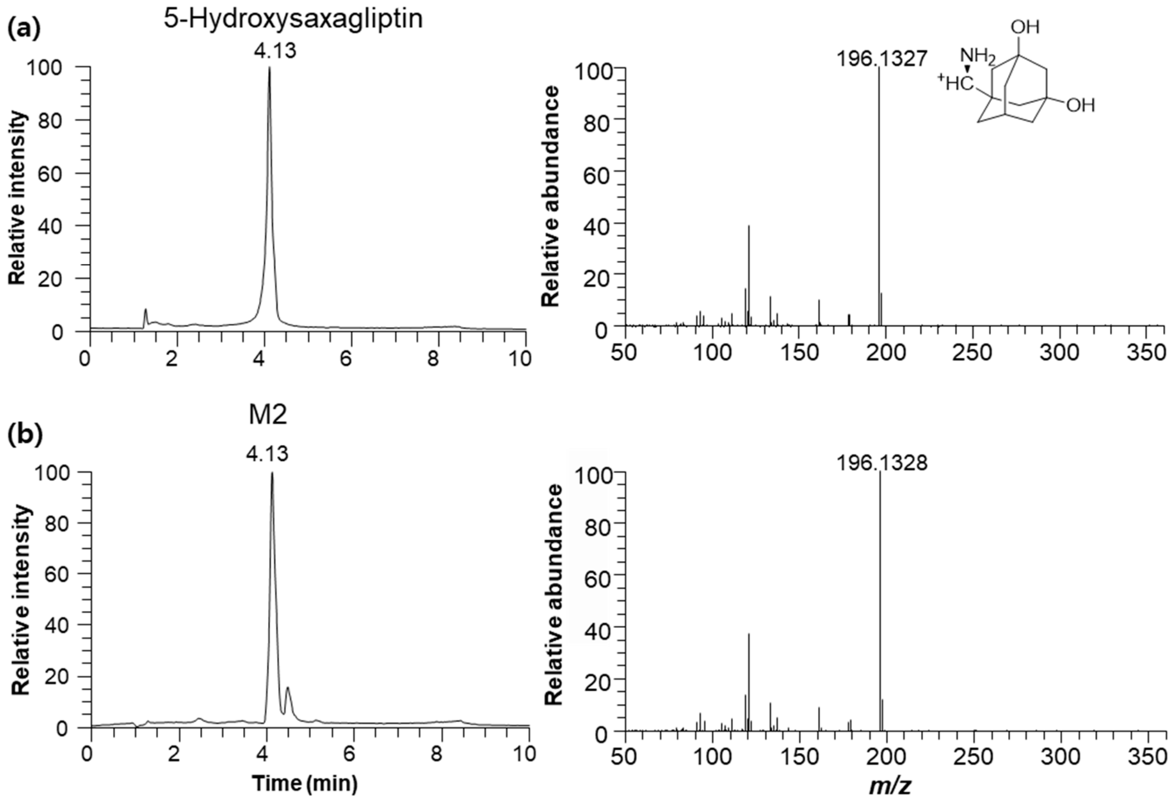

Figure S1. Representative extracted ion chromatograms (*m*/*z* 332.1969, left panel) and product ion scan mass spectra (right panel) of the authentic 5-hydroxysaxagliptin standard (a) and M2 (b).

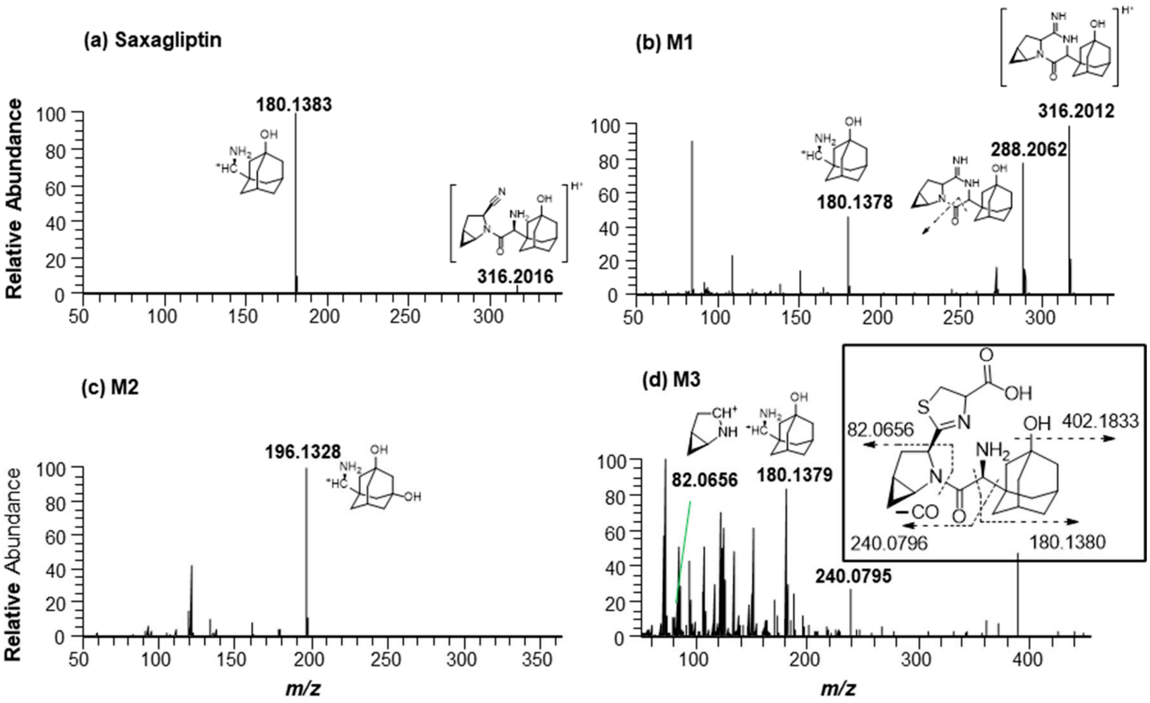

Figure S2. Product ion scan mass spectra of saxagliptin (P, a, *m/z* 316.2020), M1 (b, *m/z* 316.2020), M2 (c, *m/z* 332.1969), and M3 (d, *m/z* 420.1952) which were found in bile, plasma, or liver tissues following the intraperitoneal injection of saxagliptin in rats.

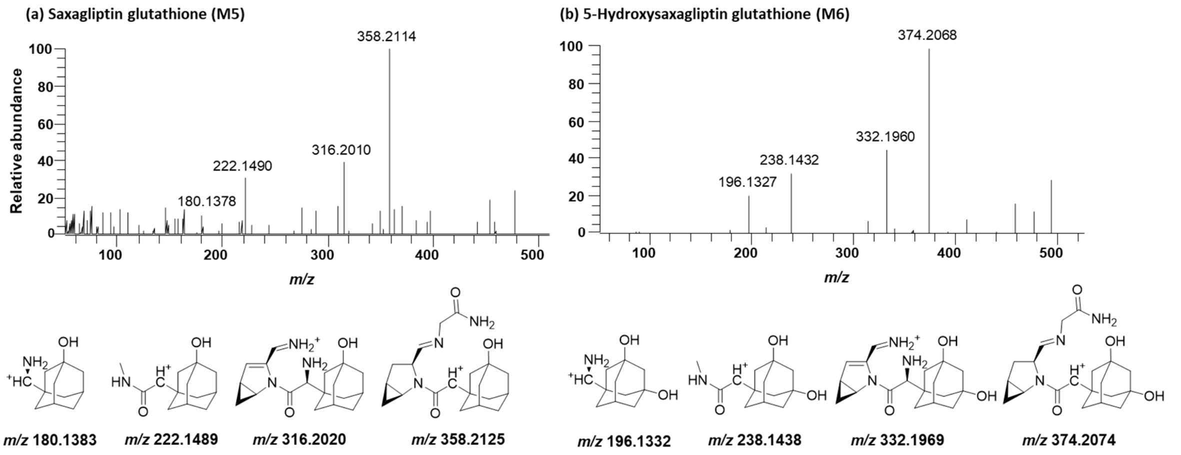

Figure S3. Product ion scan mass spectra of saxagliptin–cysteinylglycine conjugate (a, *m*/*z* 477.2166, M5) and 5-hydroxysaxagliptin–cysteinylglycine conjugate (b, *m*/*z* 493.2115, M6) and proposed chemical fragment ion structure.

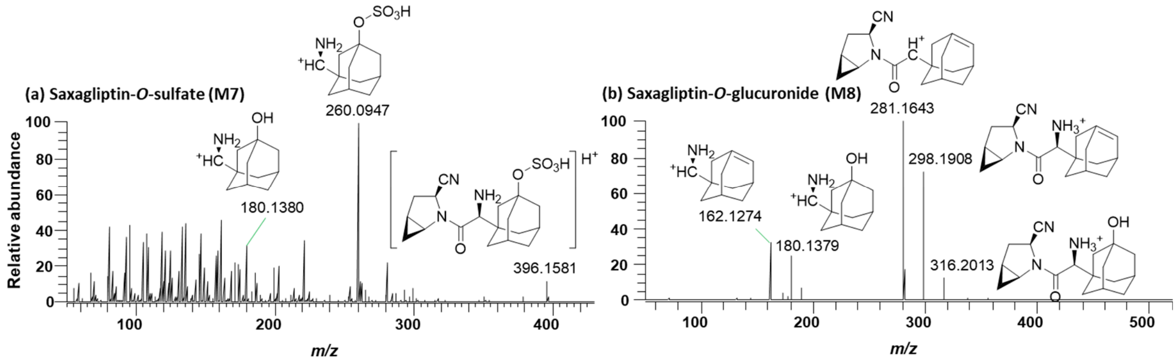

Figure S4. Product ion scan mass spectra of saxagliptin-O-sulfate (a, *m*/*z* 396.1588, M7) and saxagliptin-O-glucuronide (b, *m*/*z* 492.2340, M8) and the proposed chemical structure of the fragment ions.
